# Supplementary material for: NFYA promotes malignant behavior of triple-negative breast cancer in mice through the regulation of lipid metabolism
Source: Commun Biol. 2023 Jun 2;6:596. doi: 10.1038/s42003-023-04987-9 (PMC10238388; doi:10.1038/s42003-023-04987-9)
Supplement: Supplementary file 2 — Supplementary Information [file 42003_2023_4987_MOESM2_ESM.pdf]

Supplementary Information

NFYA promotes malignant behavior of triple-negative breast cancer in mice through the regulation of lipid metabolism

Nobuhiro Okada[1,2,*], Chihiro Ueki[1], Masahiro Shimazaki[3], Goki Tsujimoto[1], Susumu Kohno[4], Hayato Muranaka[4,5], Kiyotsugu Yoshikawa[6], Chiaki Takahashi[4]

[1]Graduate School of Interdisciplinary Science & Engineering in Health Systems, Okayama University, Okayama 700-8530, Japan.

[2]Department of Pharmacology, Kyoto Prefectural University of Medicine, Kyoto 602-8566, Japan.

[3]Laboratory for Malignancy Control Research, Medical Innovation Center, Kyoto University, Kyoto 606-8501, Japan.

[4]Division of Oncology and Molecular Biology, Cancer Research Institute, Kanazawa University, Kanazawa 920-1192, Japan.

[5]Samuel Oschin Cancer Center, Cedars-Sinai Medical Center, Los Angeles, CA 90048, USA.

[6]Faculty of Pharmaceutical Sciences, Doshisha Women's College of Liberal Arts, Kyoto 610-0395, Japan.

*Corresponding author: okadan@koto.kpu-m.ac.jp

a

Sperm	Egg	No. electropolated	No. survived	No. 2-cell	2-cell %	No. transferred (female mice)	No. birth	Birth %
C57BL/6J	C57BL/6J	50	50	50	100 %	50 (3)	27	54.0 %

b

	Number of founder (F0) mice			
	Nfyav1$^{+/+}$	*Nfyav1*$^{+/-}$	*Nfyav1*$^{-/-}$	N.D.
Male	6	2	3	2
Female	4	0	4	2
Total	10	2	7	4

N.D.; not determined

Supplementary Table 1 Generation of Nfyav1 knockout mice. **a** Table shows the information on mutation rates and production statistics following the introduction of sgRNAs and Cas9 into zygotes. **b** Genotypes of F0 mice.

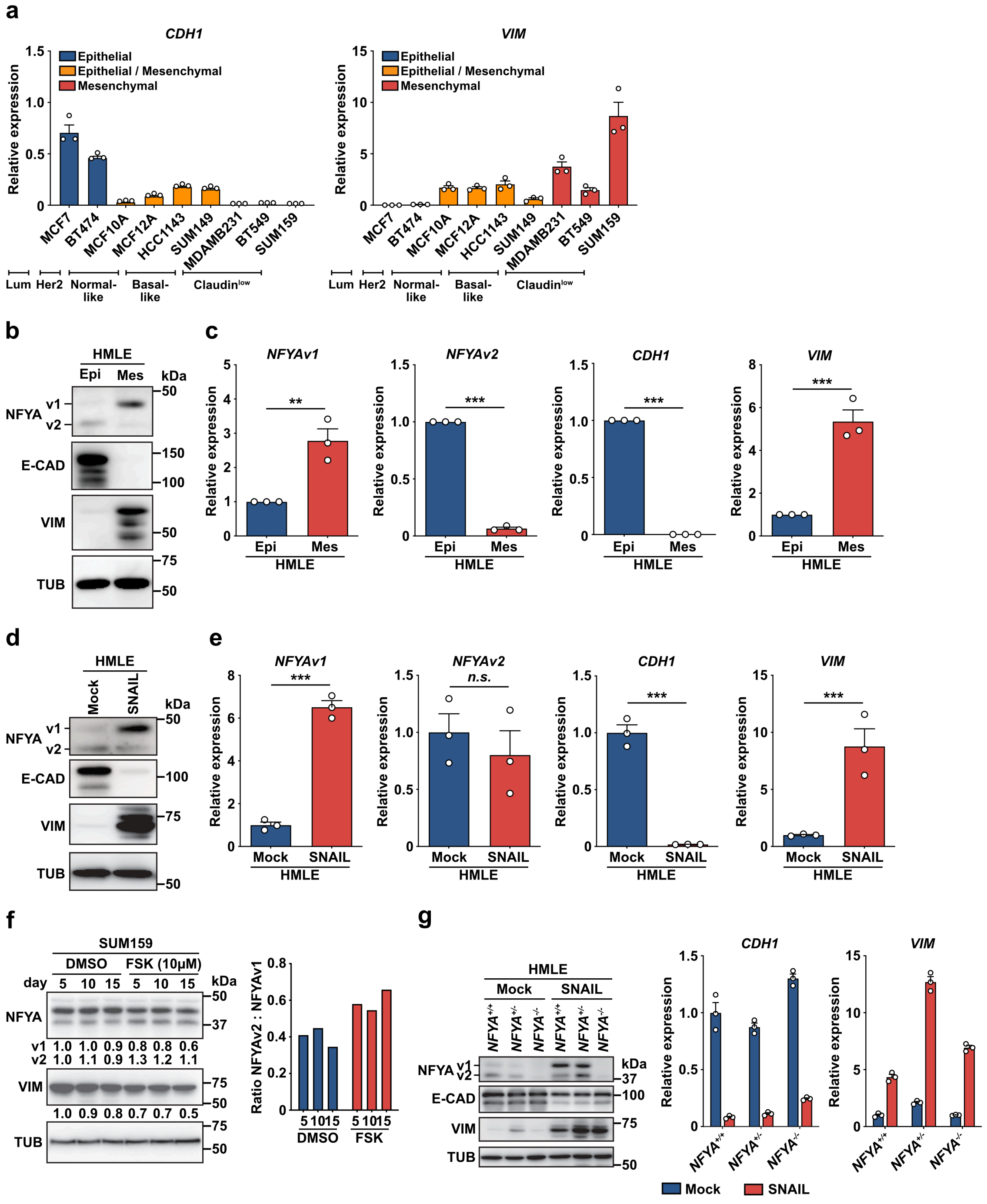

Supplementary Figure 1 NFYA switches the expression of alternative splicing variants during EMT progression. **a** qRT-PCR analysis of epithelial marker gene (*CDH1,* left panel) and mesenchymal marker gene (*VIM,* right panel) mRNA levels in various breast cancer cell lines. N=3 biologically independent experiments. **b, c** Western blot (**b**) and qRT-PCR analysis (**c**) of NFYAv1 and NFYAv2 in HMLE-Epi and HMLE-Mes cells. E-CAD/CDH1 and VIM are markers for epithelial and mesenchymal cells, respectively. N=3 biologically independent experiments for qRT-PCR. **d, e** Western blot (**d**) and qRT-PCR analysis (**e**) of NFYAv1 and

NFYAv2 in HMLE cells overexpressed SNAIL to induce EMT. E-CAD/CDH1 and VIM are markers for epithelial and mesenchymal cells, respectively. N=3 biologically independent experiments for qRT-PCR. **f** MET induced by 10 μM of Forskolin (FSK) treatment in SUM159 cells induced reverse switching from NFYAv1 to NFYAv2 expression. The ratio of each band is relative to the amount of each protein in DMSO treatment for 5 days. The graph shows the ratio of NFYAv2/NFYAv1. **g** Western blot and qRT-PCR analysis in *NFYA*$^{+/+}$, *NFYA*$^{+/-}$, and *NFYA*$^{-/-}$ HMLE cells with or without SNAIL overexpression. N=3 biologically independent experiments for qRT-PCR. All error bars represent SEM; (*n.s.*) not significant; (**) P<0.01; (***) P<0.001.

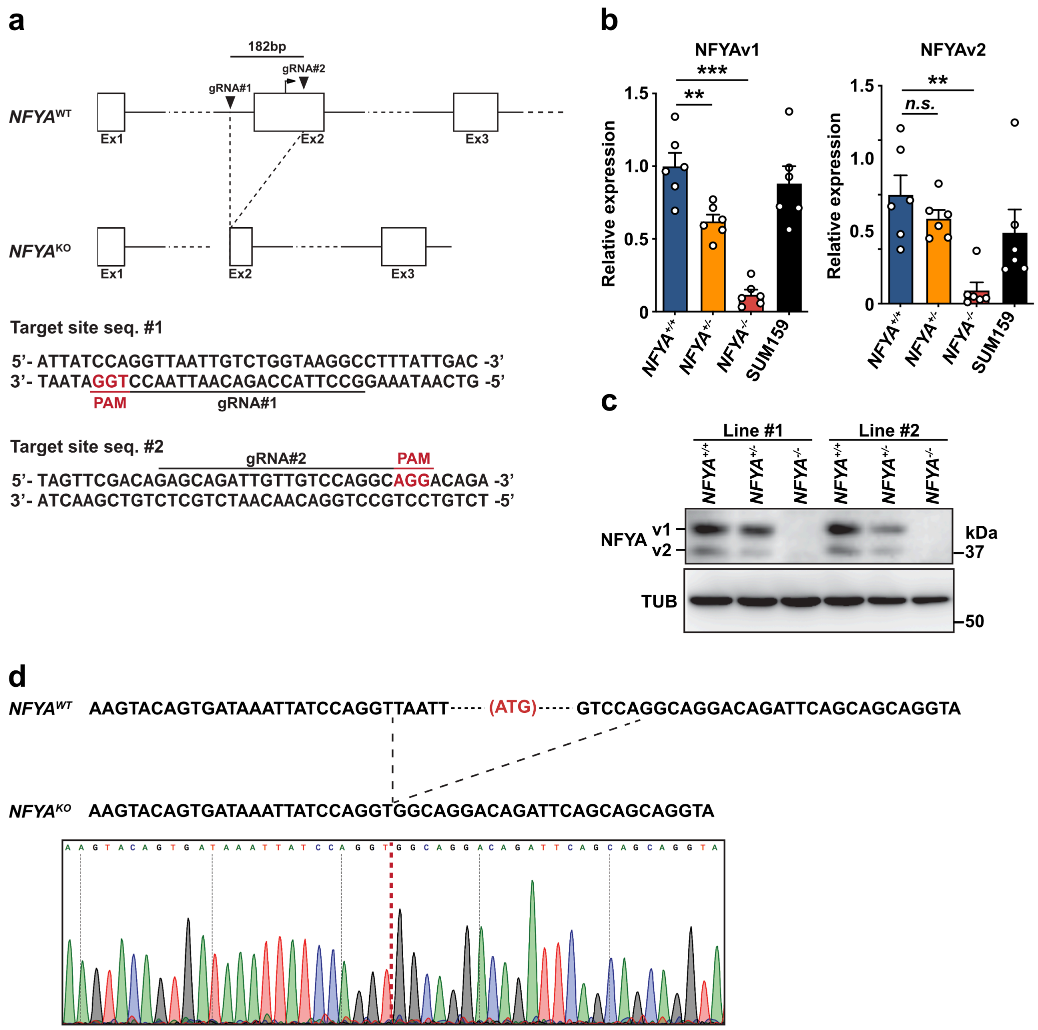

Supplementary Figure 2 Generation of NFYA-deficient SUM159 cells. **a** Schematic diagram of two sgRNA target sites located on both sides of the start codon and sequences of the selected sites for editing *NFYA*. Protospacer-adjacent motif (PAM) sequences are highlighted in red. **b, c** qRT-PCR (**b**) and western blot analysis (**c**) to validate the deletion of *NFYA* gene expression generated by CRISPR/Cas9 in SUM159 cells. N=6 biologically independent experiments for qRT-PCR. **d** The result of sequence analysis of *NFYA*$^{-/-}$ SUM159 cells manipulated by CRISPR/Cas9. *NFYA*$^{-/-}$ SUM159 cells deleted the start codon ATG. All error bars represent SEM; (*n.s.*) not significant; (**) P<0.01; (***) P<0.001.

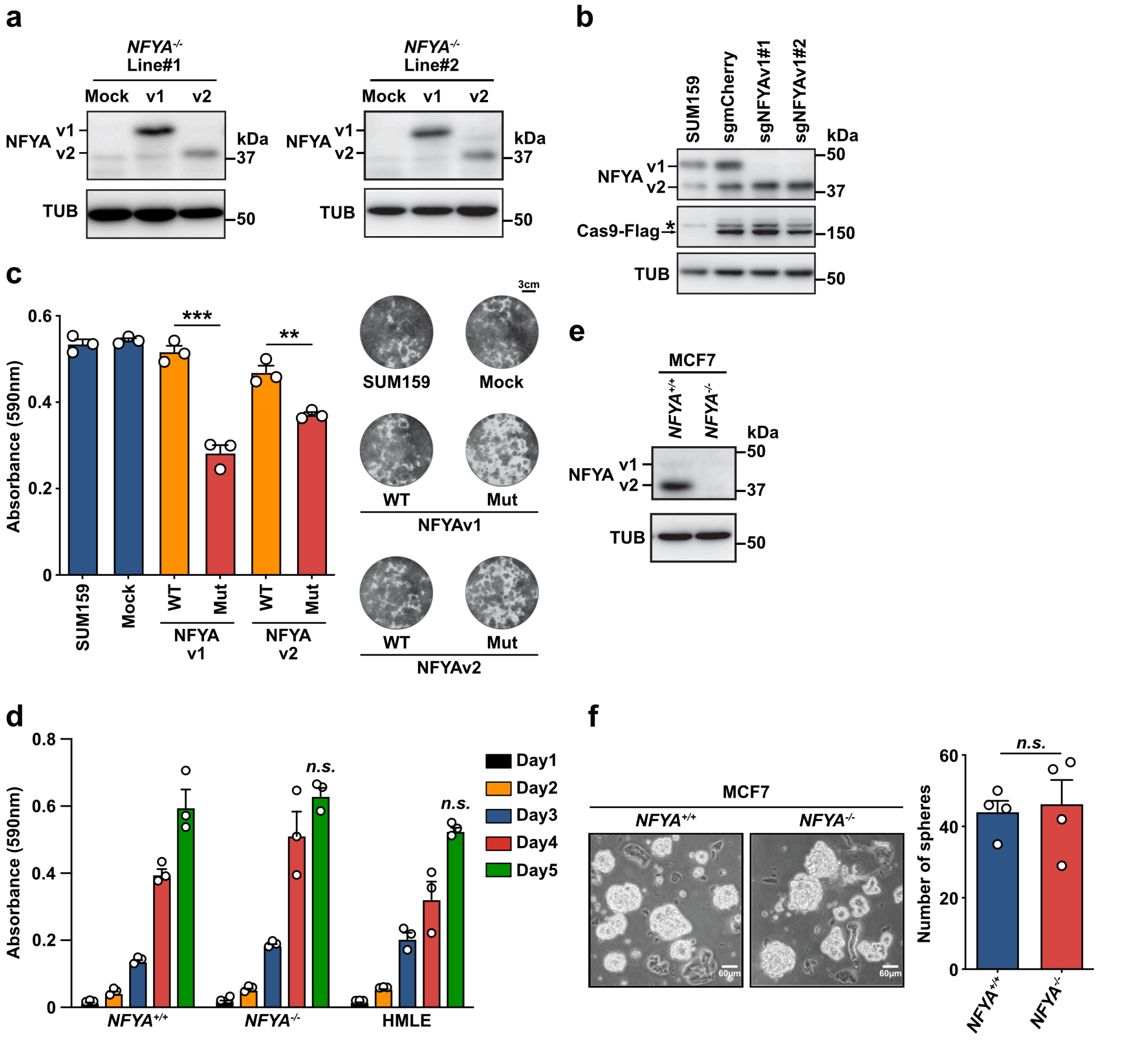

Supplementary Figure 3 NFYAv1 deficiency inhibits tumour cell growth and tumorigenesis in TNBCs. **a** The validation of overexpression of NFYA variants in *NFYA*$^{-/-}$ SUM159 cells. **b** *NFYAv1* specific knockout was validated by western blot analysis. Asterisk indicates nonspecific bands. sgNFYAv1#1 and sgNFYAv1#2 target different sequences. sgmCherry used as a control targeted the sequence of mCherry. **c** Quantification and representative pictures of 0.5 % crystal violet staining of SUM159 cells overexpressed WT and dominant-negative mutants of both NFYA variants. N=3 biologically independent experiments. Scale bar indicates 3 cm. **d** A bar graph shows the quantification of 0.5 % crystal violet staining of *NFYA*$^{+/+}$ and *NFYA*$^{-/-}$ HMLE cells. N=3 biologically independent experiments. **e** Western blot analysis to validate the deletion of *NFYA* gene expression generated by CRISPR/Cas9 in MCF7 cells. **f** Representative images of sphere formation by *NFYA*$^{+/+}$ and *NFYA*$^{-/-}$ MCF7 cells. A bar graph shows the number of spheres larger than 60 μm. N=4 biologically independent experiments. Scale bars indicate 60 μm. All error bars represent SEM; (*n.s.*) not significant; (**) P<0.01; (***) P<0.001.

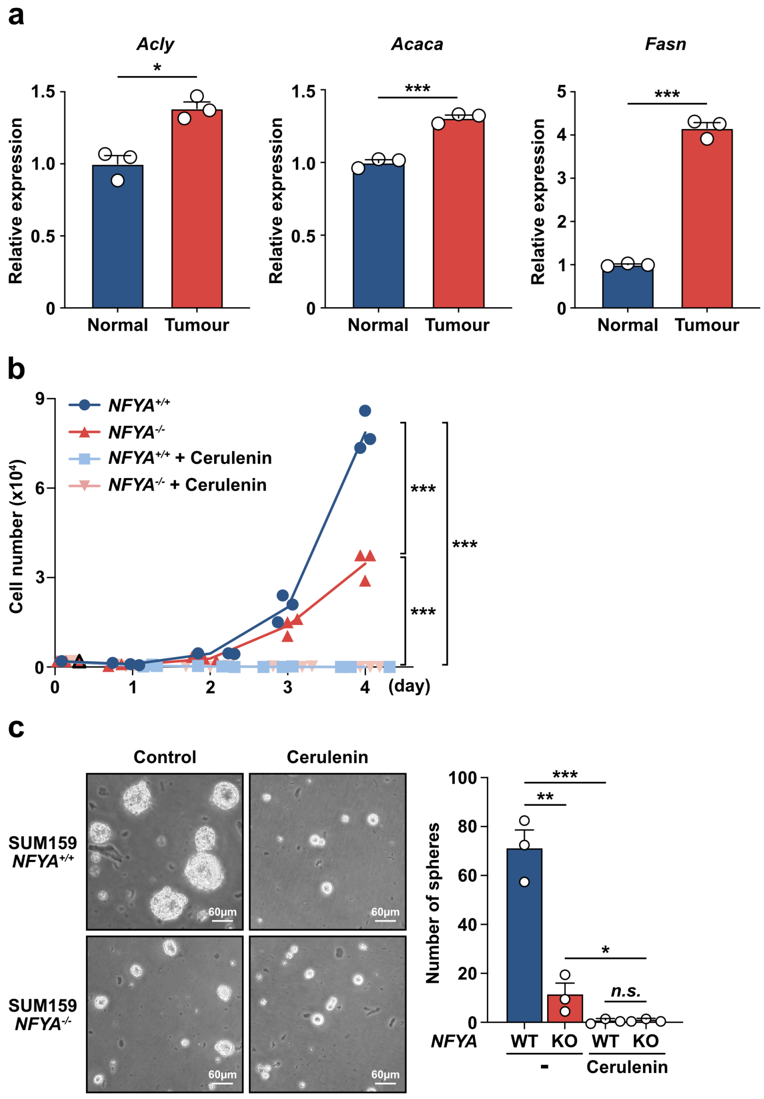

Supplementary Figure 4 Lipogenesis is essential for the regulation of TNBCs malignant behavior. **a** qRT-PCR analysis of *Acly*, *Acaca*, and *Fasn* in breast cancer cells (primary cells isolated from mouse breast cancer tissue) compared with non-transformed mouse mammary epithelial cells (NMuMG cells). N=3 biologically independent experiments for qRT-PCR. **b** Cumulative population of cells was measured for 4 consecutive days in *NFYA*$^{+/+}$ and *NFYA*$^{-/-}$ SUM159 cells with or without the addition of cerulenin. N=3 biologically independent experiments. **c** Representative images of sphere formation by *NFYA*$^{+/+}$ and *NFYA*$^{-/-}$ SUM159 cells with or without the addition of cerulenin. A bar graph shows the number of spheres larger than 60 μm in each group. N=3 biologically independent experiments. Scale bars indicate 60 μm. All error bars represent SEM; (*n.s.*) not significant; (*) P<0.05; (**) P<0.01; (***) P<0.001.

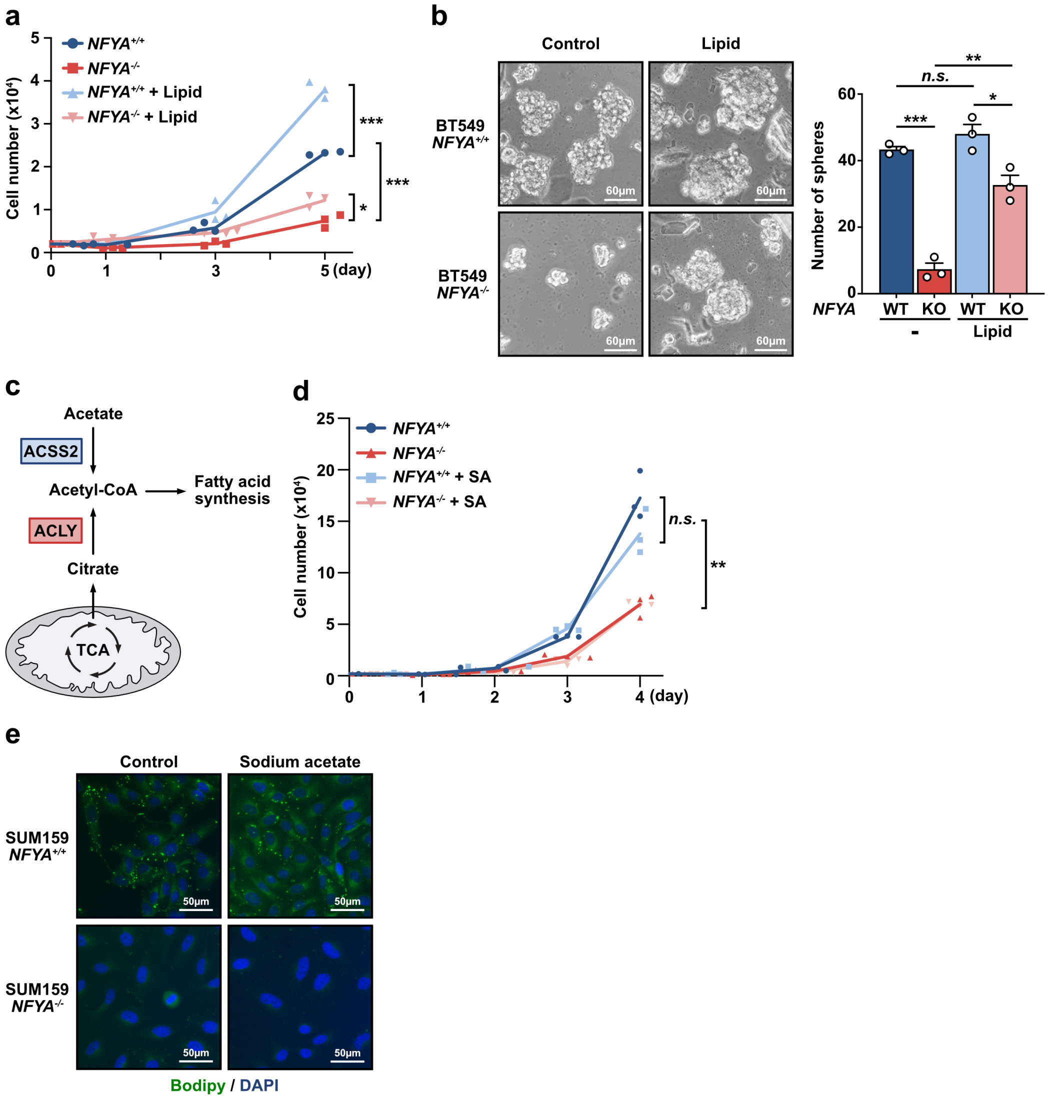

Supplementary Figure 5 NFYA regulates lipid metabolism for malignant behavior of TNBCs. **a** Cumulative population of cells was measured every other day for 5 days in *NFYA*$^{+/+}$ and *NFYA*$^{-/-}$ BT549 cells with or without adding a lipid mixture. N=3 biologically independent experiments. **b** Representative images of sphere formation by *NFYA*$^{+/+}$ and *NFYA*$^{-/-}$ BT549 cells with or without the addition of lipid mixture. A bar graph shows the number of spheres larger than 60 μm in each group. N=3 biologically independent experiments. Scale bars indicate 60 μm. **c** A schematic diagram depicting two different pathways for acetyl-CoA synthesis. **d** Cumulative population of cells was measured for 4 consecutive days in *NFYA*$^{+/+}$ and *NFYA*$^{-/-}$ SUM159 cells treated with or without sodium acetate. N=3 biologically independent experiments. **e** Representative fluorescence images of lipid droplet (green) detected with Bodipy 493/503 and nucleus (blue) detected with DAPI in *NFYA*$^{+/+}$ and *NFYA*$^{-/-}$ SUM159 cells treated with or without sodium acetate. Scale bars indicate 50 μm. All error bars represent SEM; (*n.s.*) not significant; (*) P<0.05; (**) P<0.01; (***) P<0.001.

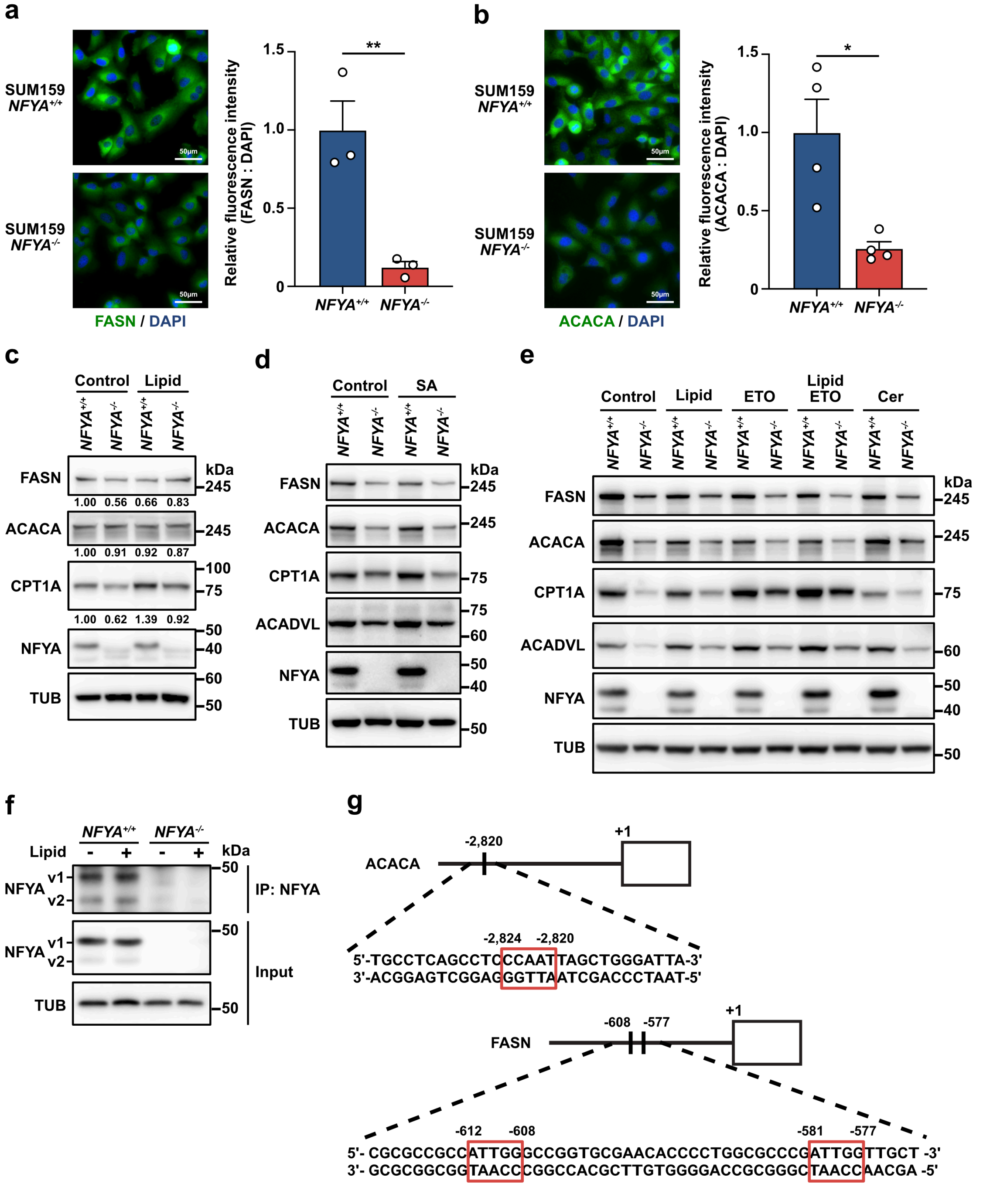

Supplementary Figure 6 NFYA enhances lipogenesis by transcriptional activation of ACACA and FASN. **a, b** *NFYA*$^{+/+}$ and *NFYA*$^{-/-}$ SUM159 cells were immunostained with anti-FASN (**a**, green) or anti-ACACA (**b**, green) antibodies and counterstained with DAPI for DNA (blue). A bar graph shows the relative fluorescence intensity of FASN or ACACA staining to DAPI. N=3 for FASN and N=4 for ACACA biologically independent experiments. Scale bars indicate 50 μm. **c** Western blot analysis of the expression levels of differentially expressed genes in *NFYA*$^{+/+}$ and *NFYA*$^{-/-}$ BT549 cells treated with or without lipid mixture. **d** Western blot analysis

of lipogenesis and FAO-related genes in *NFYA*$^{+/+}$ and *NFYA*$^{-/-}$ SUM159 cells treated with or without sodium acetate (1 mM for 3 days). **e** Western blot analysis of lipogenesis and FAO-related genes in *NFYA*$^{+/+}$ and *NFYA*$^{-/-}$ SUM159 cells treated with or without lipid mixture, etomoxir (ETO), both, and cerulenin. **f** Western blot analysis validated immunoprecipitation of NFYA by using the anti-NFYA antibody in *NFYA*$^{+/+}$ and *NFYA*$^{-/-}$ SUM159 cells. **g** The location of NFYA binding sites on ACACA and FASN promoter. All error bars represent SEM; (*) $P<0.05$; (**) $P<0.01$.

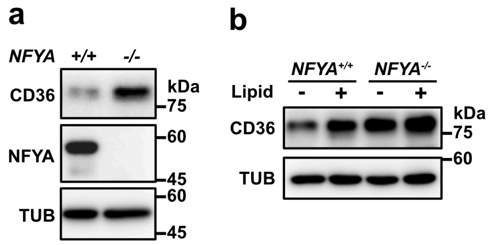

Supplementary Figure 7 NFYA deficiency and the addition of lipids increase CD36 expression. **a, b** Western blot analysis of fatty acid translocase (CD36) in *NFYA*$^{+/+}$ and *NFYA*$^{-/-}$ SUM159 cells without (a) or with (b) lipid addition.

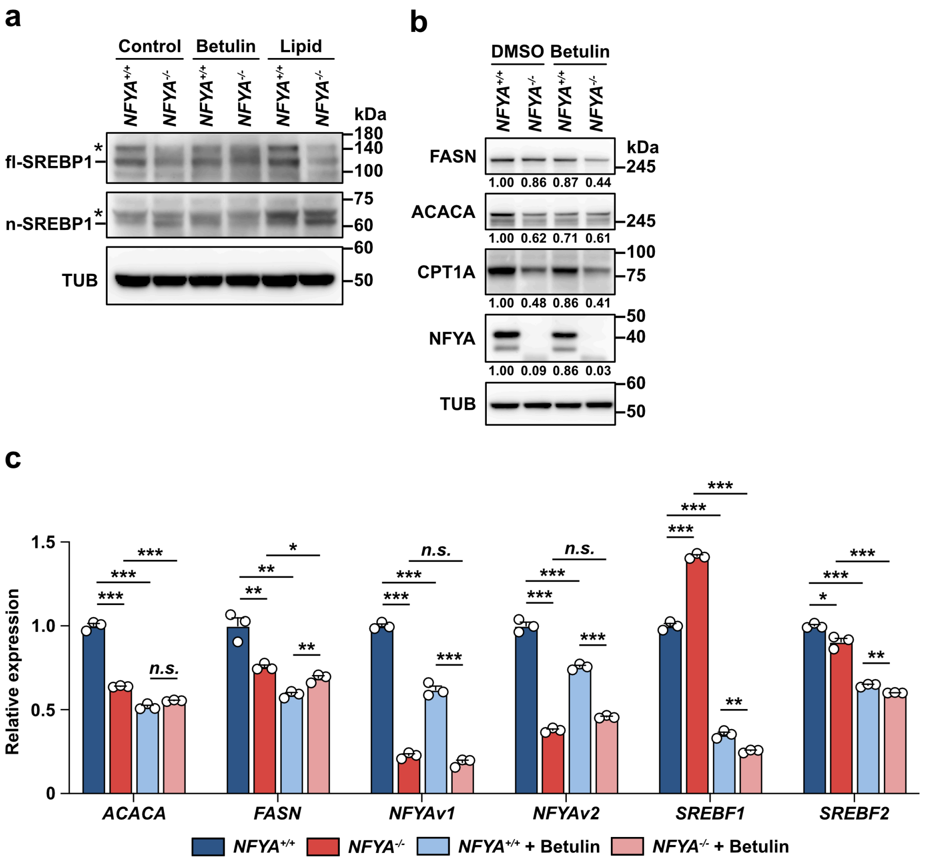

Supplementary Figure 8 NFYA and SREBP1 cooperatively regulate the expression of lipogenic enzymes. **a** Western blot analysis of the expression levels of SREBP1 in *NFYA*$^{+/+}$ and *NFYA*$^{-/-}$ SUM159 cells treated with or without betulin or lipid mixture. fl-SREBP1; full-length SREBP1, n-SREBP1; nuclear SREBP1. Asterisk indicates nonspecific bands. **b, c** Western blot (b) and qRT-PCR analysis (c) of the expression levels of differentially expressed genes in *NFYA*$^{+/+}$ and *NFYA*$^{-/-}$ SUM159 cells treated with or without betulin. N=3 biologically independent experiments for qRT-PCR. All error bars represent SEM; (*n.s.*) not significant; (*) P<0.05; (**) P<0.01; (***) P<0.001.

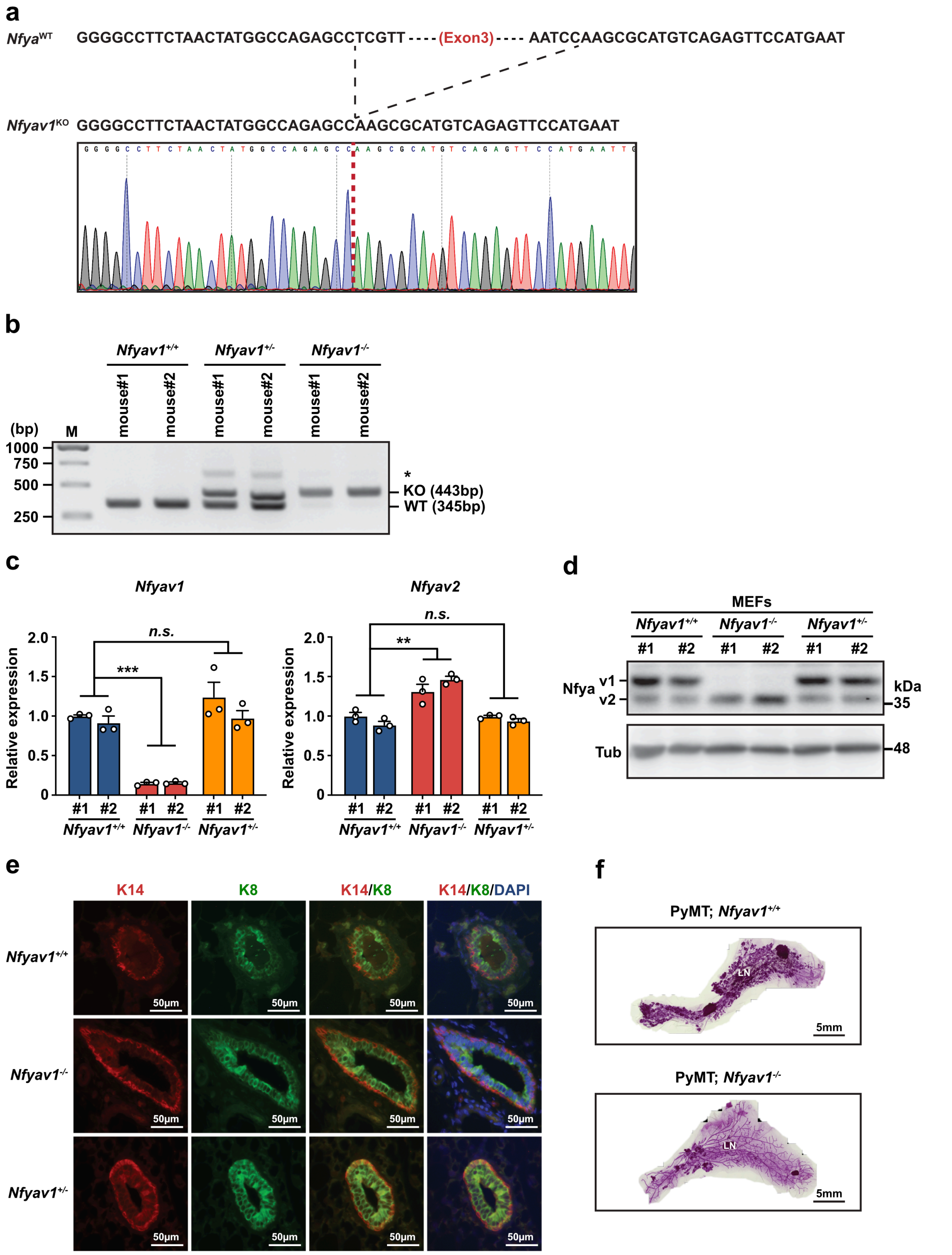

Supplementary Figure 9 Nfyav1 enhances tumorigenesis via the regulation of Acaca and Fasn expression *in vivo*. **a** The sequence analysis of *Nfyav1*$^{-/-}$ mouse manipulated by CRISPR/Cas9. **b** PCR analysis of genomic DNA from tails of *Nfyav1*$^{+/+}$, *Nfyav1*$^{+/-}$, and *Nfyav1*$^{-/-}$ mice using primers shown in Fig. 5A. Amplification products correspond to WT and KO alleles (345bp and 443bp, respectively). Asterisk indicates nonspecific bands. **c,**

d Confirming loss of Nfyav1 expression in *Nfyav1*$^{-/-}$ MEFs. Littermate-controlled *Nfyav1*$^{+/+}$, *Nfyav1*$^{+/-}$, and *Nfyav1*$^{-/-}$ MEFs were analyzed by qRT-PCR (**c**) and western blot analysis (**d**). N=3 biologically independent experiments for qRT-PCR. Error bars represent SEM; (*n.s.*) not significant; (**) P<0.01; (***) P<0.001. **e** Immunofluorescence analysis of the K14 (red), K8 (green), and DAPI (blue) in *Nfyav1*$^{+/+}$, *Nfyav1*$^{+/-}$, and *Nfyav1*$^{-/-}$ mammary glands. Scale bars indicate 50 μm. **f** Representative images of whole-mount carmine alum staining of MMTV-PyMT; *Nfyav1*$^{+/+}$ or MMTV-PyMT; *Nfyav1*$^{-/-}$ mammary gland at 15-week-old. LN, lymph node. Scale bars indicate 5 mm.

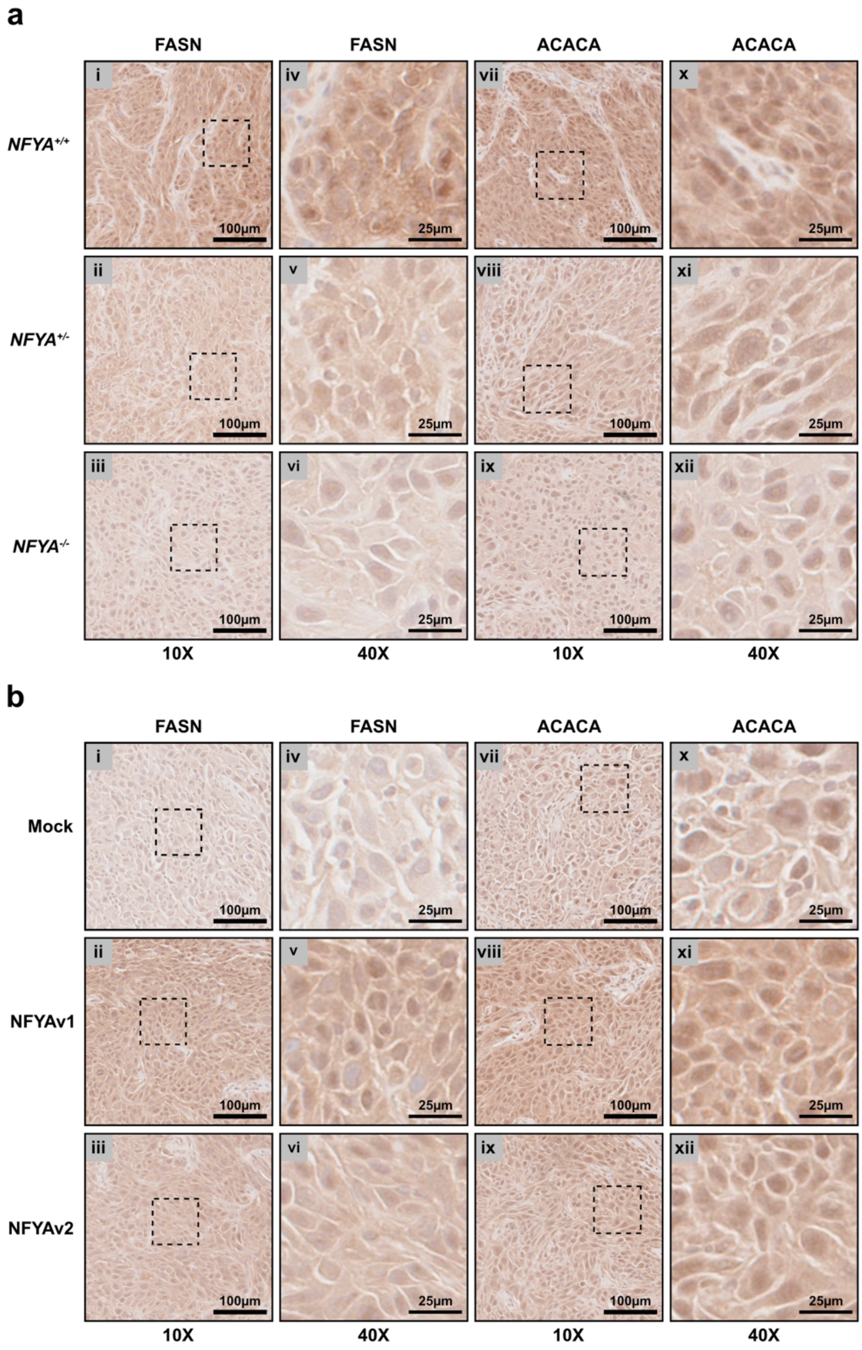

Supplementary Figure 10 The expression of FASN and ACACA is higher in tumour tissues derived from NFYAv1-expressing SUM159 cells. **a** Tumour tissue sections derived from *NFYA*$^{+/+}$, *NFYA*$^{+/-}$, and *NFYA*$^{-/-}$ SUM159 cells were stained with FASN (panels i-vi) and ACACA (panels vii-xii). **b** Tumour tissue sections derived from *NFYA*$^{-/-}$ SUM159 cells overexpressed each variant of NFYA were stained with FASN (panels i-vi) and ACACA (panels vii-xii). Scale bars: 10x images, panels i, ii, iii, vii, viii, ix, 100 μm; 40x images, panels iv, v, vi, x, xi, xii, 25 μm.

Figure 1c

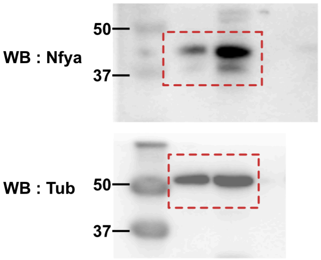

Figure 1e and Supplementary Figure 1b

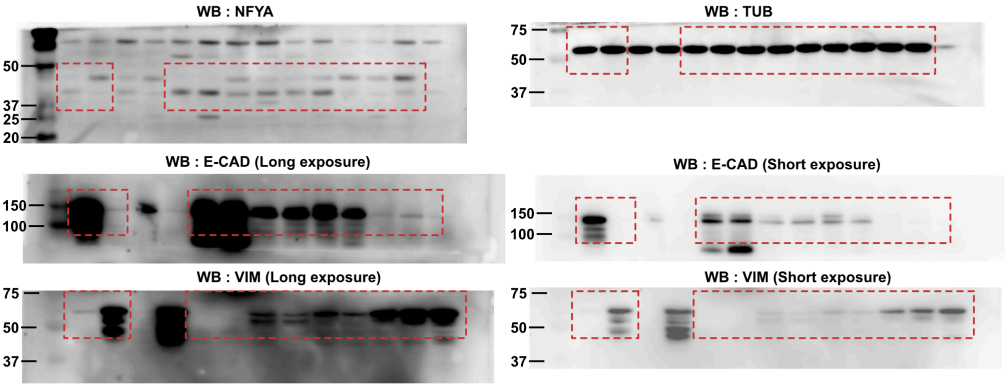

Figure 1f

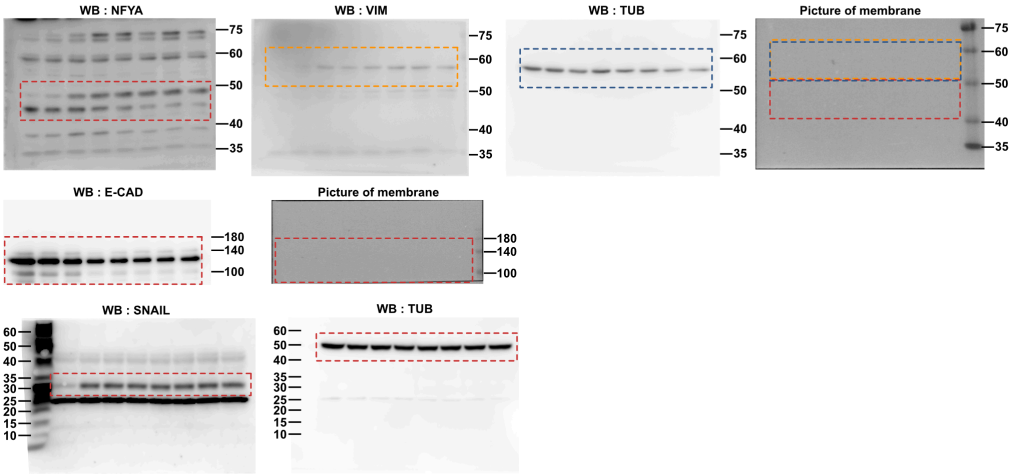

Supplementary Figure 1d

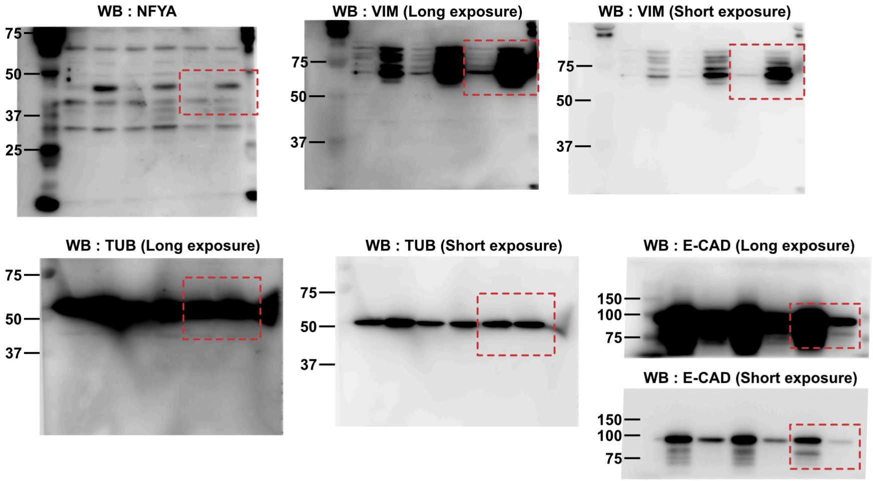

Supplementary Figure 1f

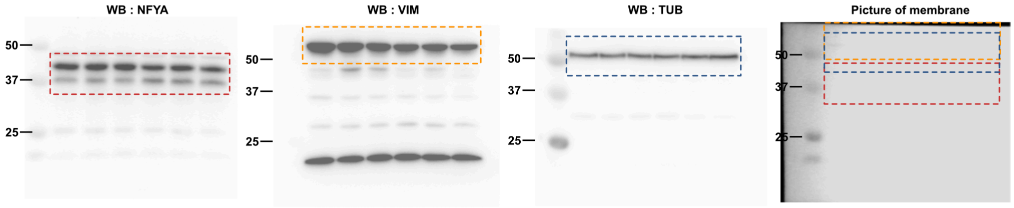

Supplementary Figure 1g

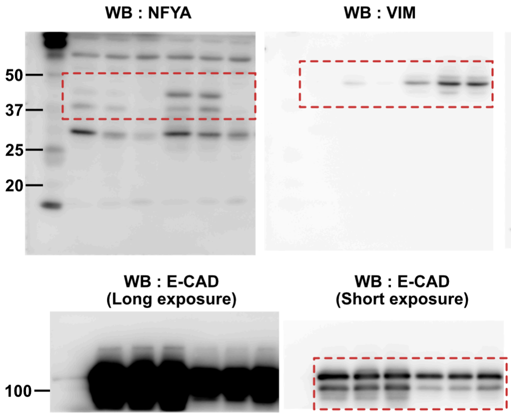

Supplementary Figure 2c

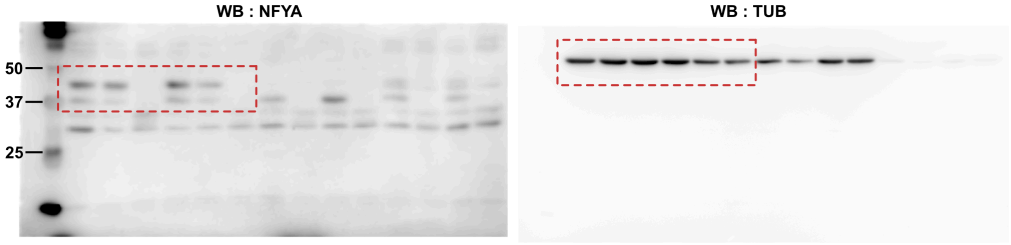

Supplementary Figure 3a

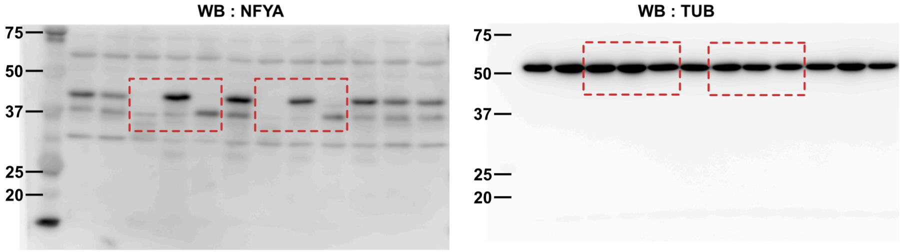

Supplementary Figure 3b

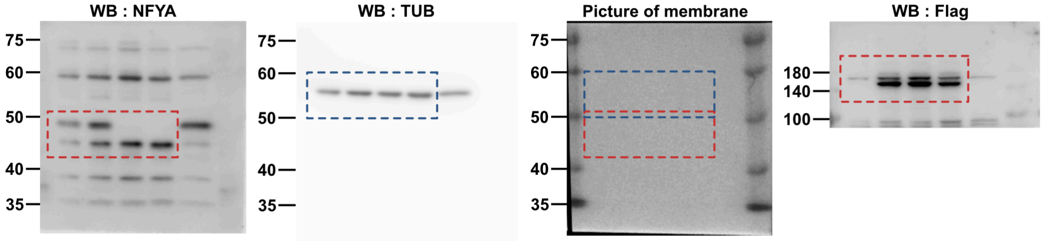

Supplementary Figure 3e

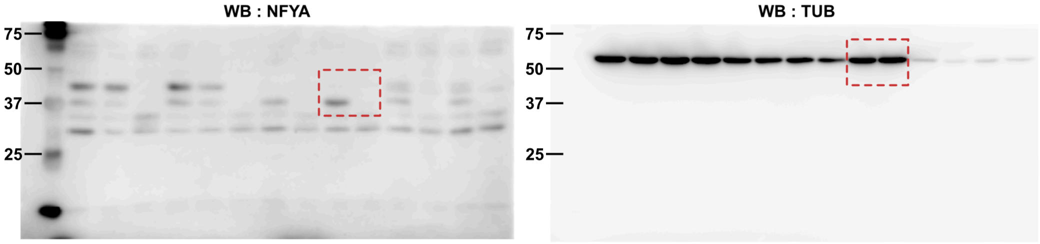

Figure 4d

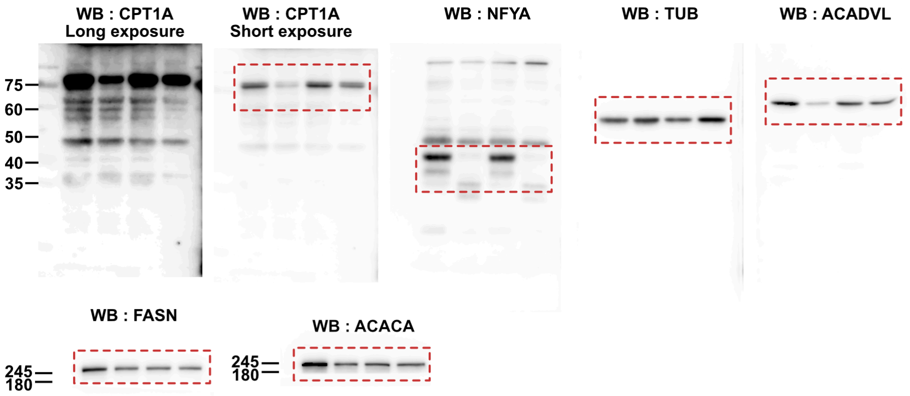

Figure 4f

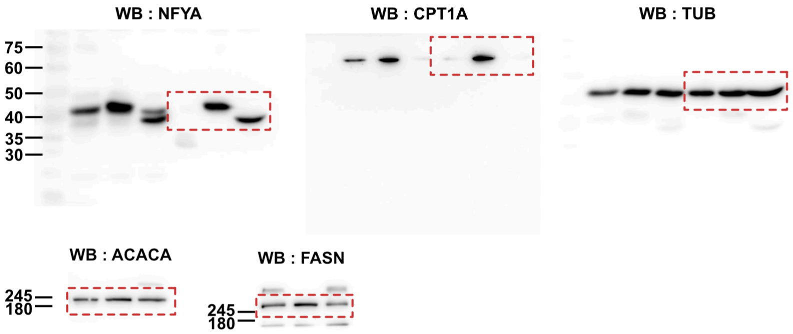

Supplementary Figure 6c

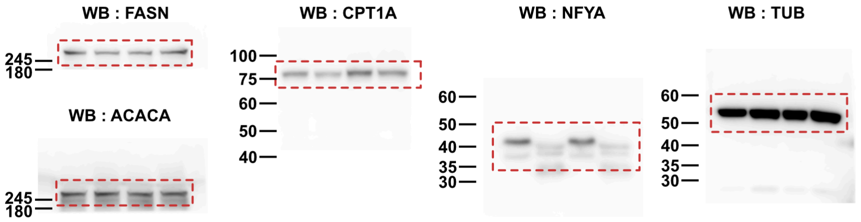

Supplementary Figure 6d

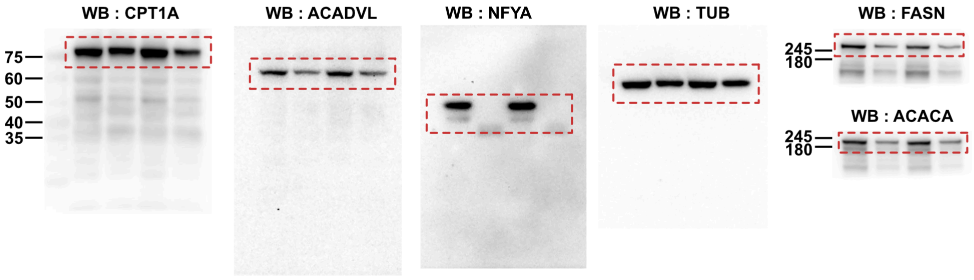

Supplementary Figure 6e

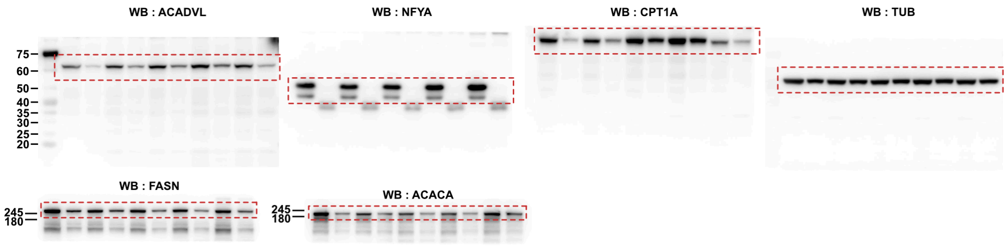

Supplementary Figure 6f

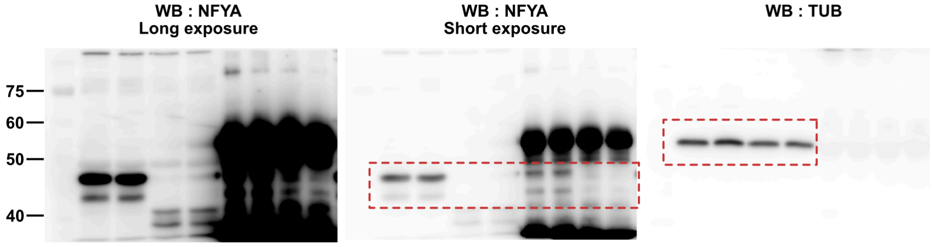

Supplementary Figure 7a

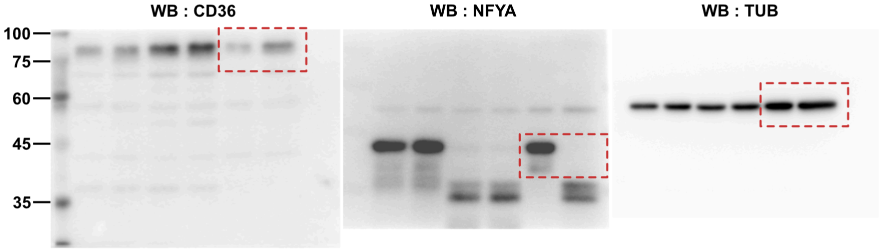

Supplementary Figure 7b

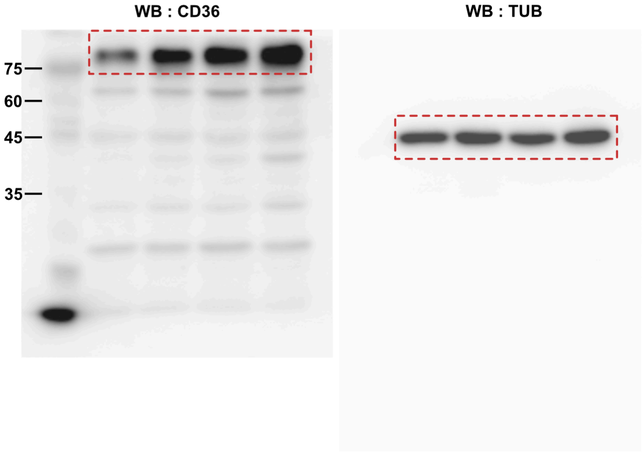

Supplementary Figure 8a

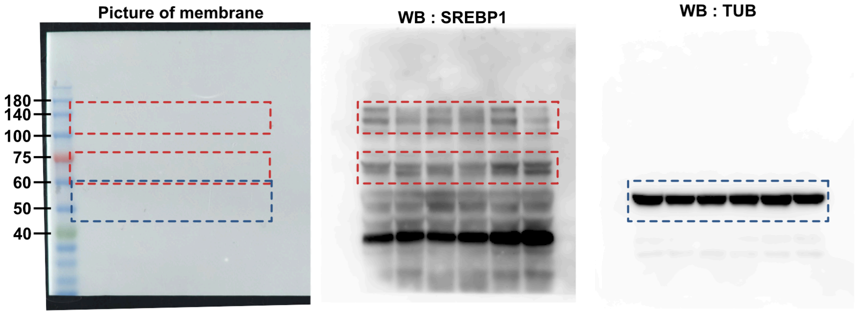

Supplementary Figure 8b

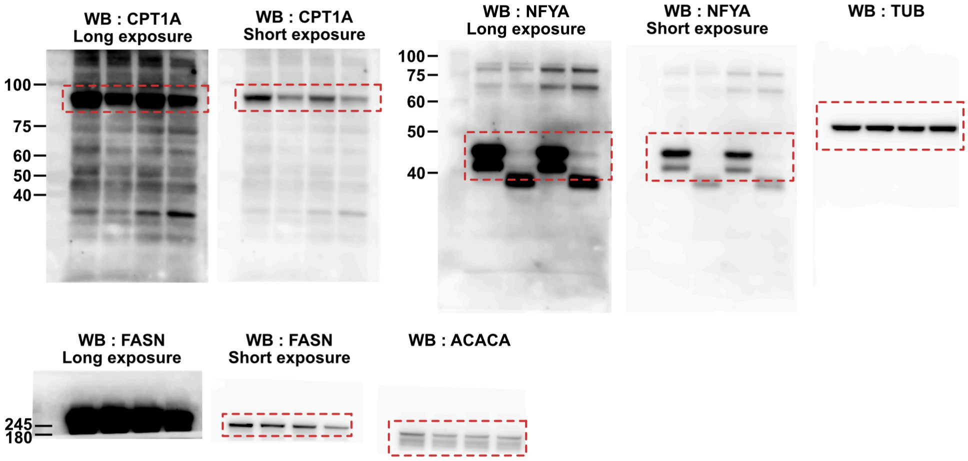

Figure 5c

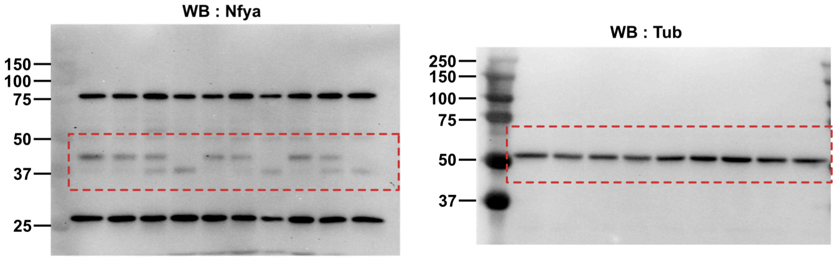

Figure 5i

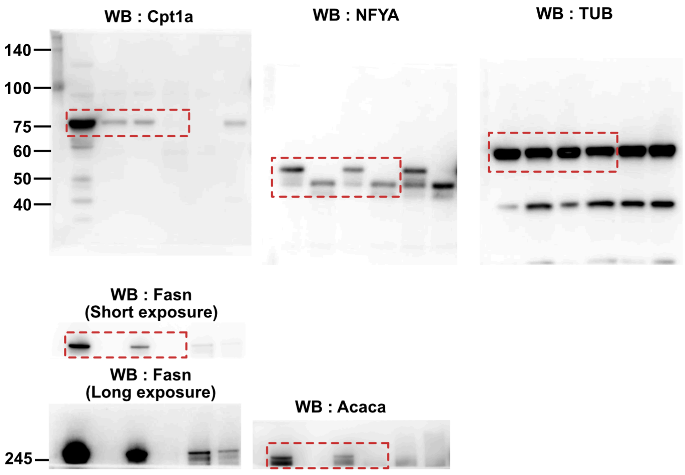

Supplementary Figure 9d

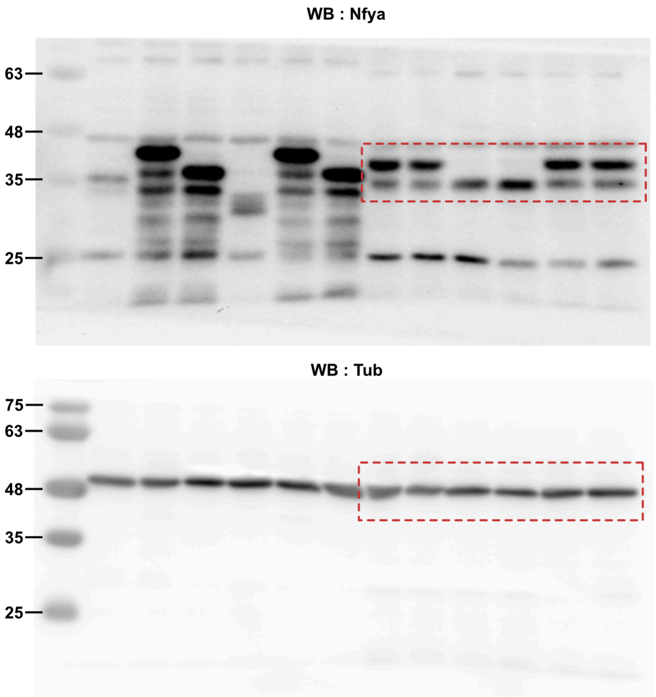

Supplementary Figure 11 Uncropped western blot images.
