# Supplementary material for: NFYA promotes malignant behavior of triple-negative breast cancer in mice through the regulation of lipid metabolism
Source: Commun Biol. 2023 Jun 2;6:596. doi: 10.1038/s42003-023-04987-9 (PMC10238388; doi:10.1038/s42003-023-04987-9)
Supplement: Supplementary file 3 — Description of Additional Supplementary Files [file 42003_2023_4987_MOESM3_ESM.pdf]

Description of Additional Supplementary Files

File name: Supplementary Data 1

Description: Information of oligonucleotides used in this study.

File name: Supplementary Data 2

Description: Numerical source data.
